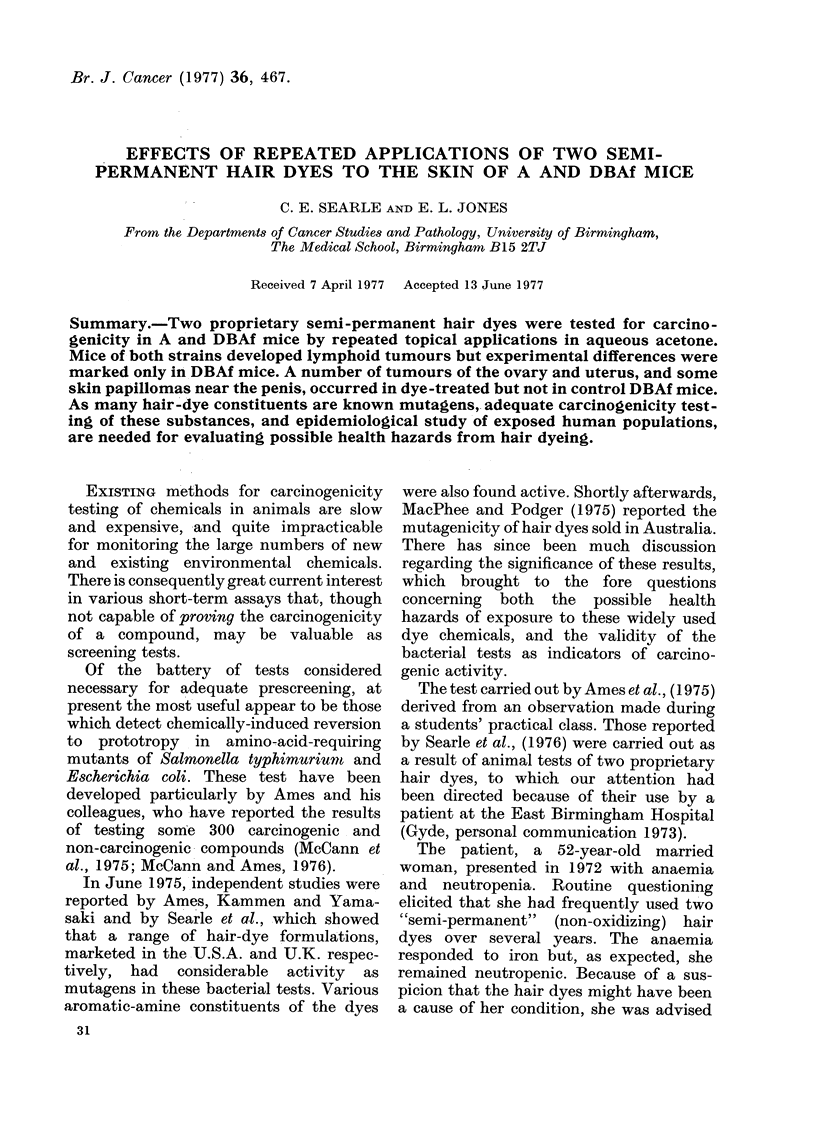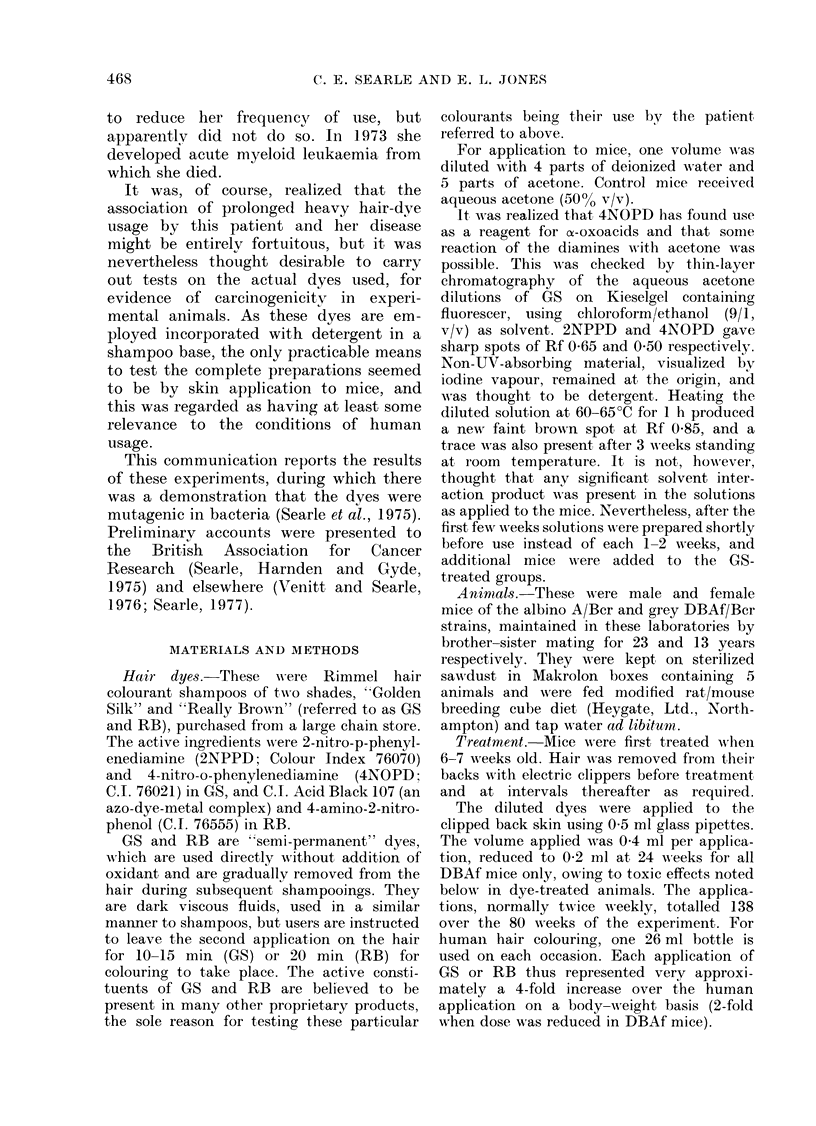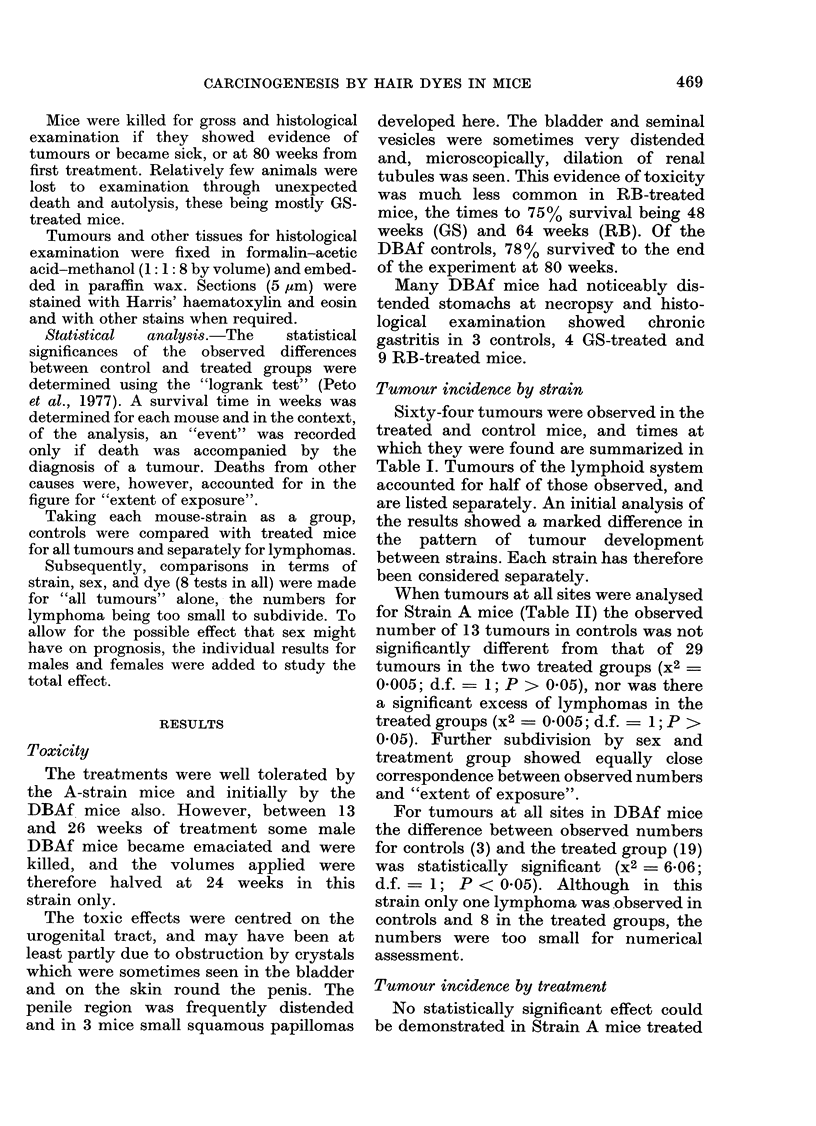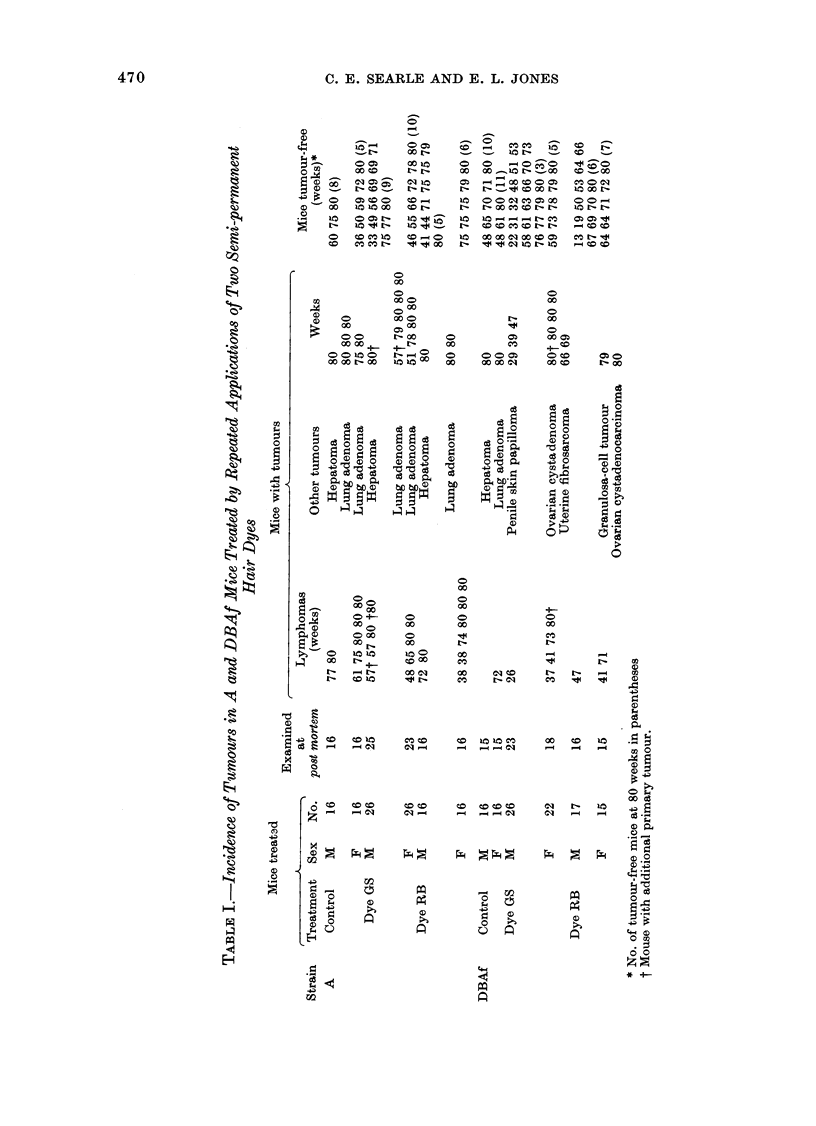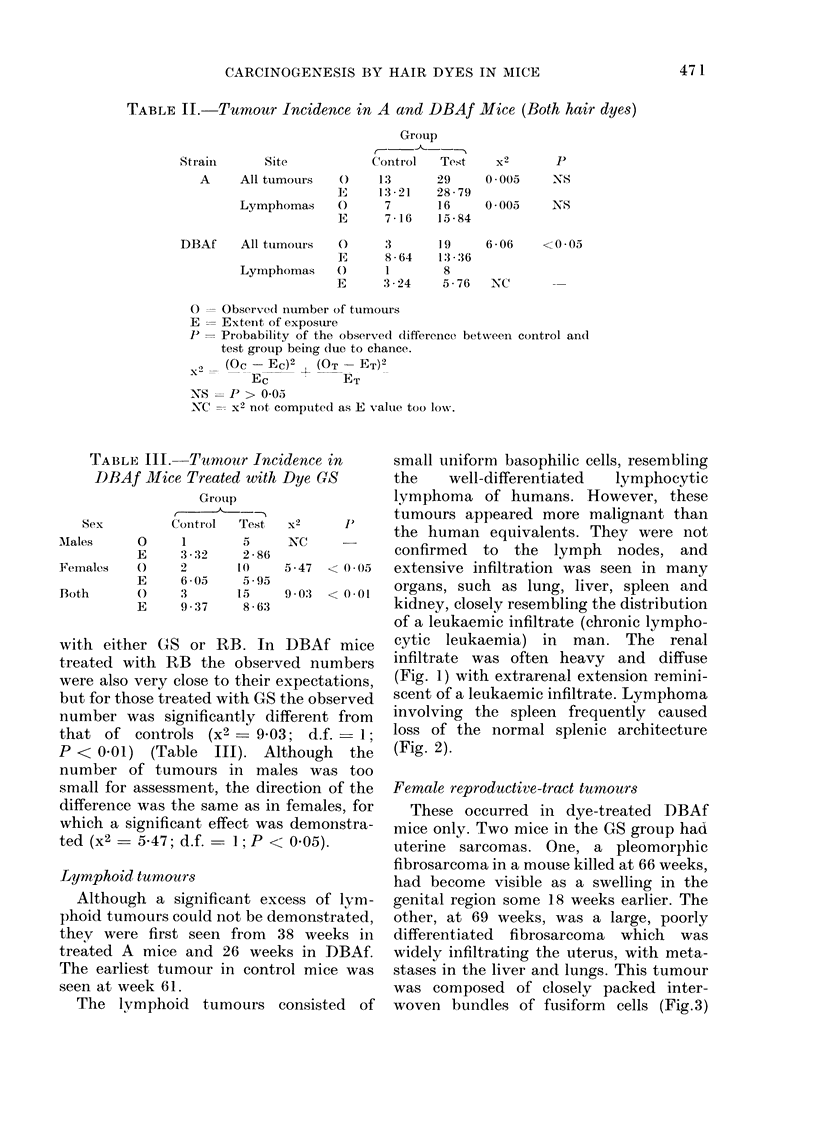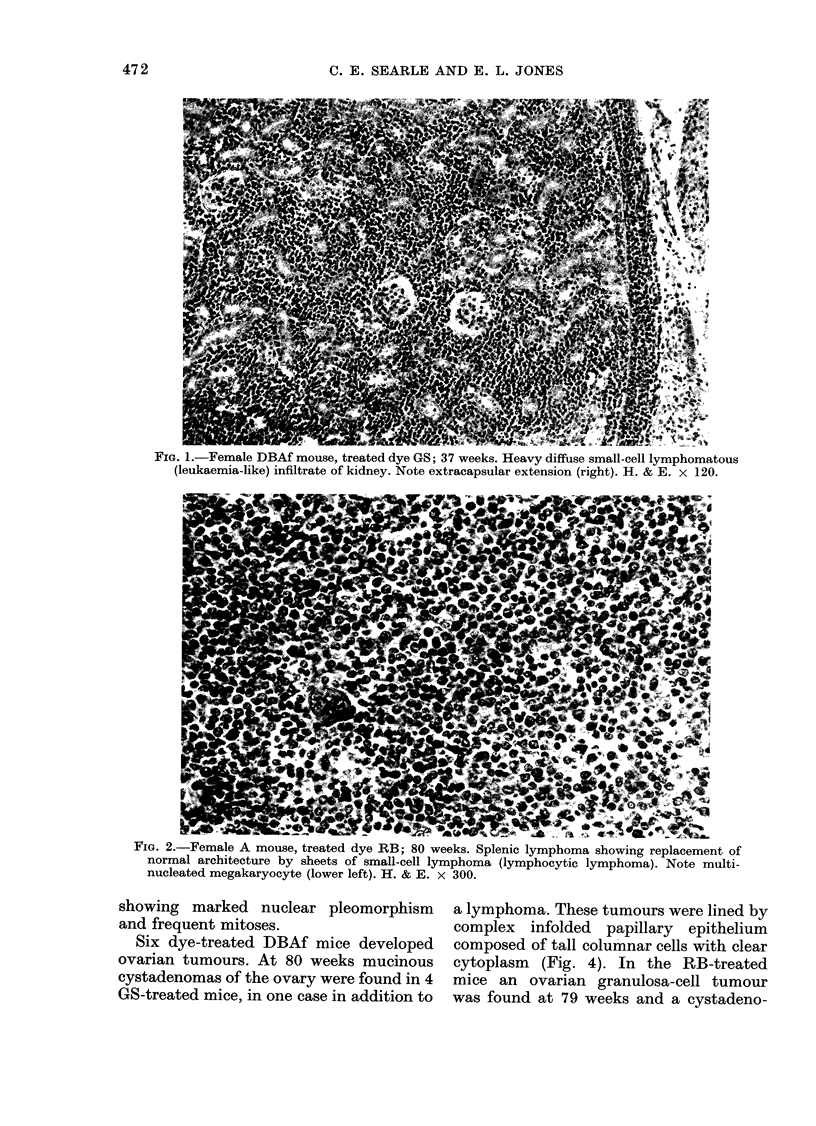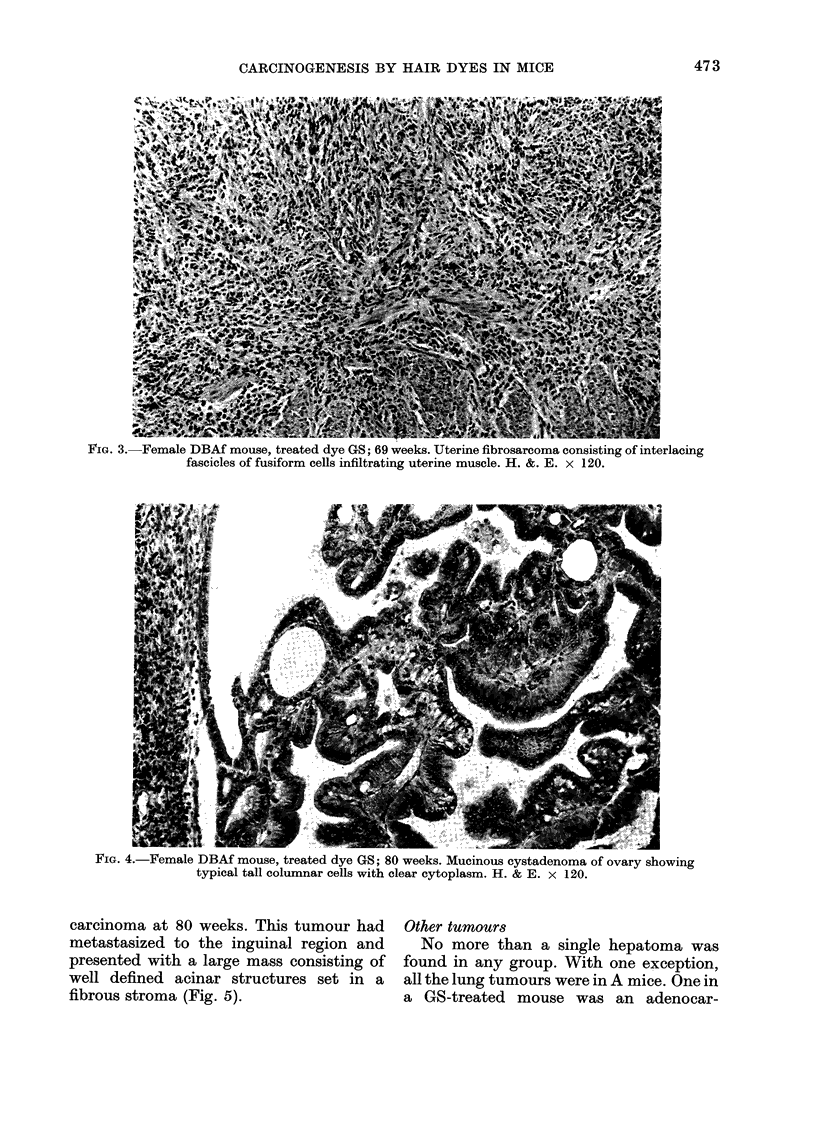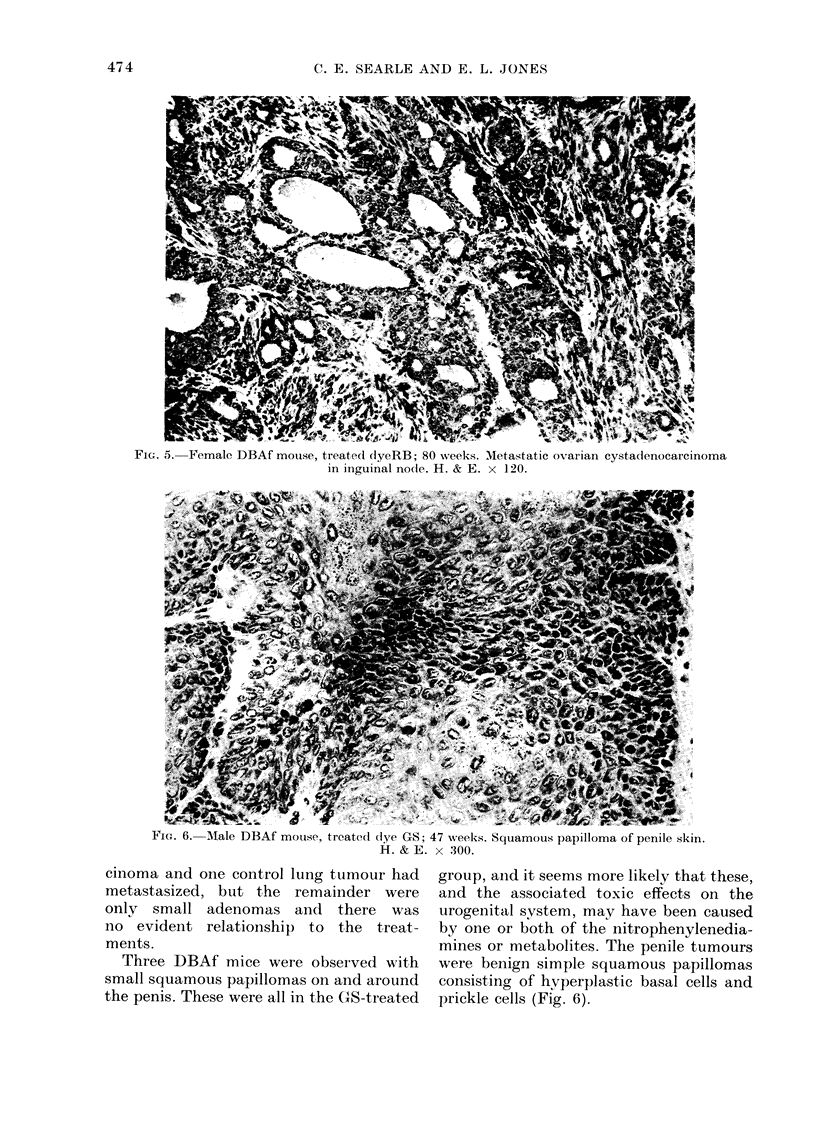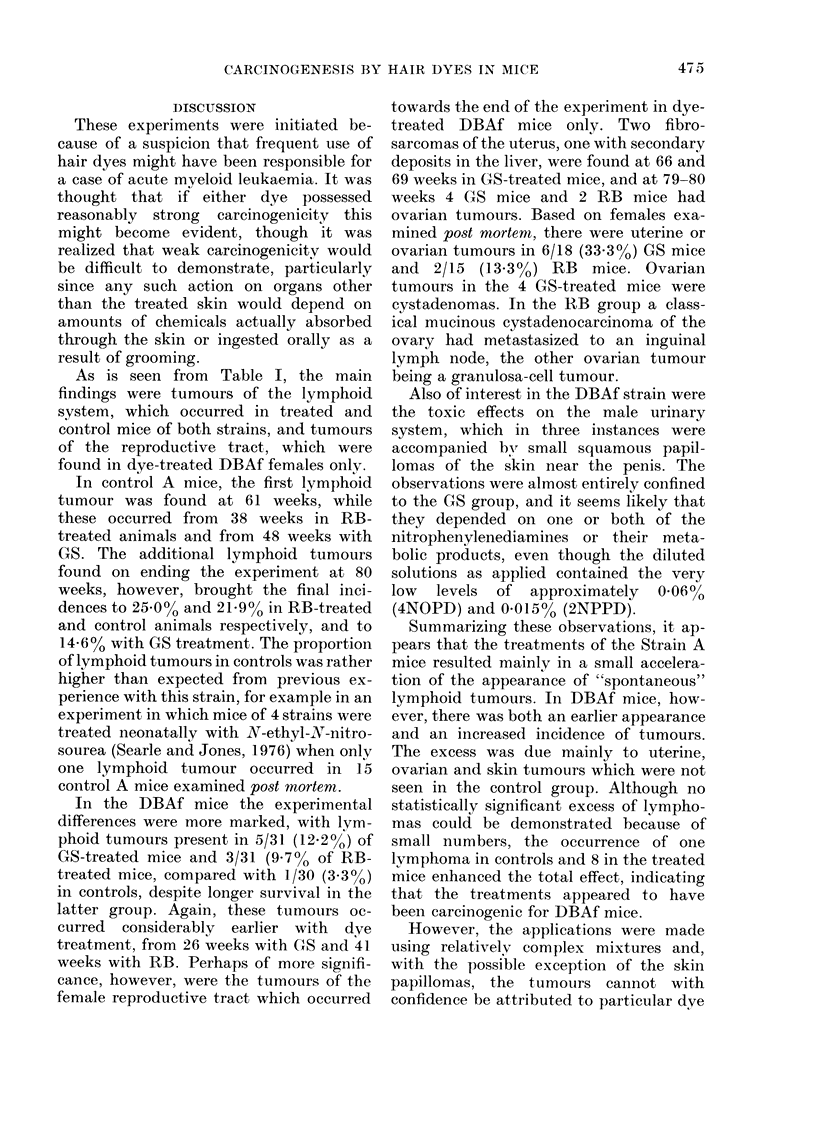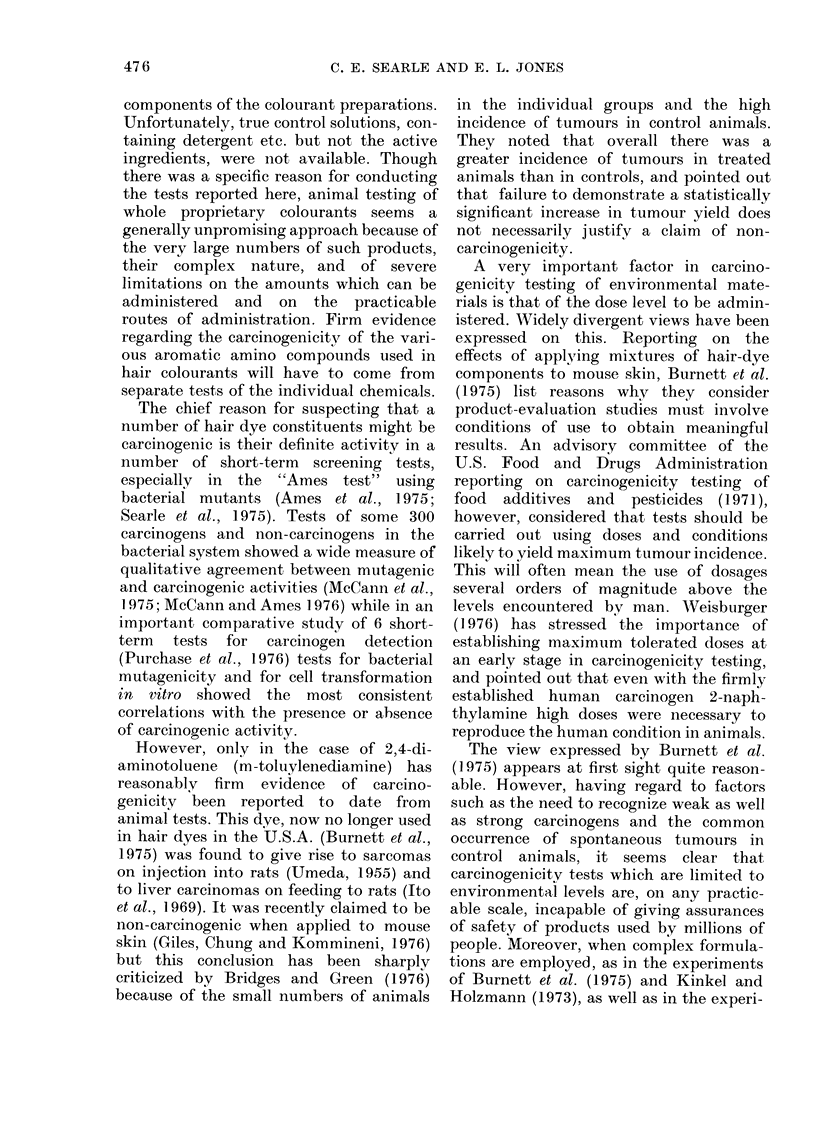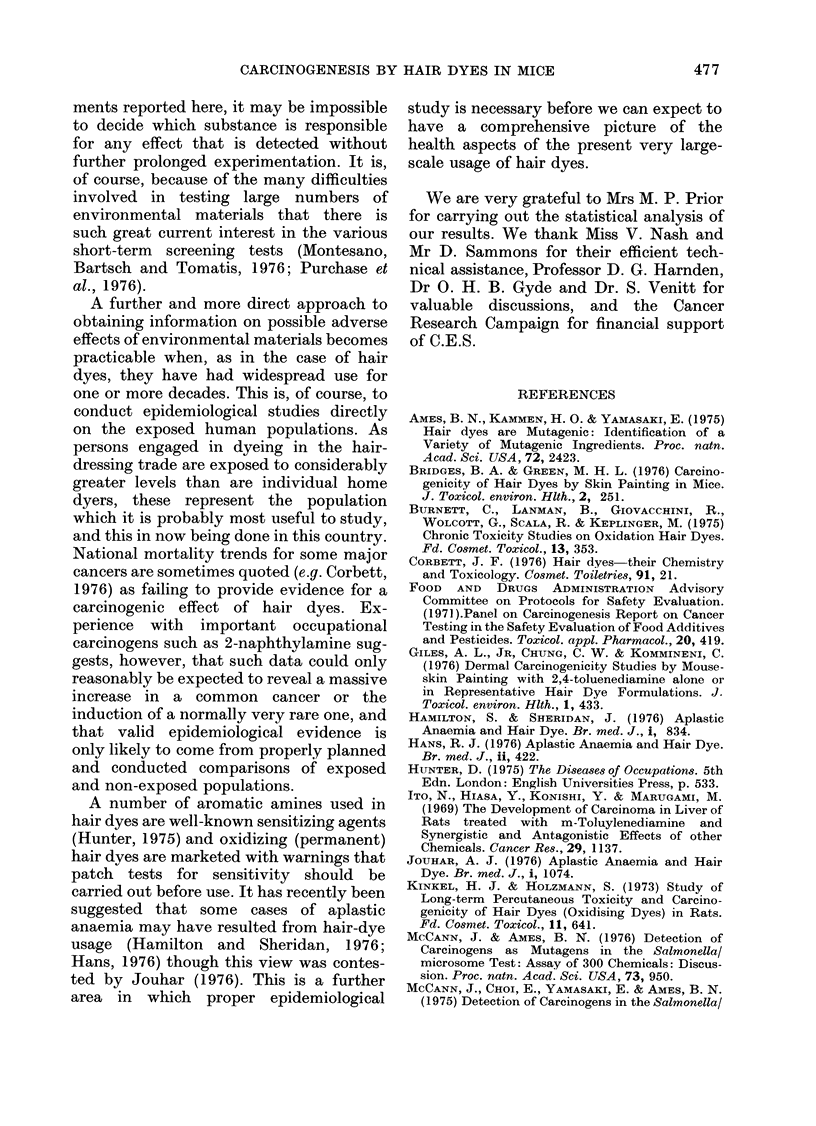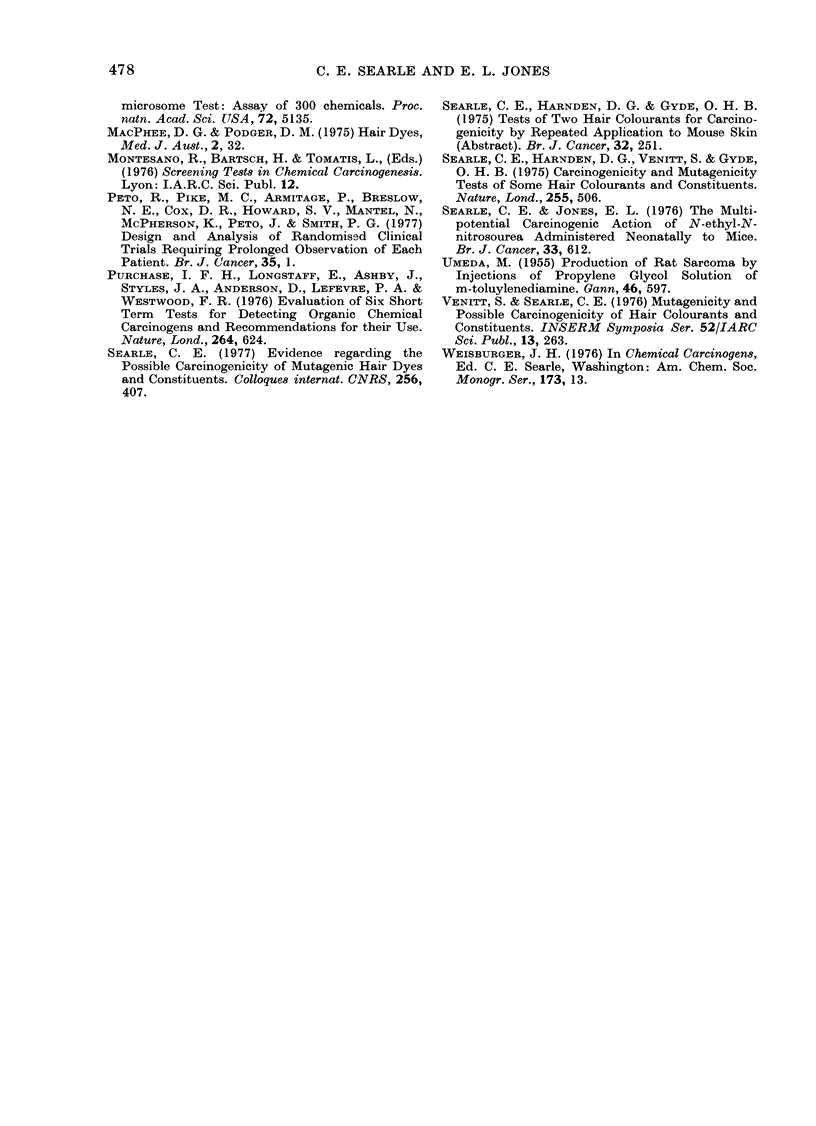# Effects of repeated applications of two semi-permanent hair dyes to the skin of A and DBAf mice.

**DOI:** 10.1038/bjc.1977.216

**Published:** 1977-10

**Authors:** C. E. Searle, E. L. Jones

## Abstract

**Images:**


*Br. J. Cancer* (1977) **36**, 467.

### EFFECTS OF REPEATED APPLICATIONS OF TWO SEMI-PERMANENT HAIR DYES TO THE SKIN OF A AND DBAf MICE

C. E. SEARLE AND E. L. JONES

*From the Departments of Cancer Studies and Pathology, University of Birmingham, The Medical School, Birmingham B15 2TJ*

Received 7 April 1977 Accepted 13 June 1977

**Summary.—Two proprietary semi-permanent hair dyes were tested for carcinogenicity in A and DBAf mice by repeated topical applications in aqueous acetone. Mice of both strains developed lymphoid tumours but experimental differences were marked only in DBAf mice. A number of tumours of the ovary and uterus, and some skin papillomas near the penis, occurred in dye-treated but not in control DBAf mice. As many hair-dye constituents are known mutagens, adequate carcinogenicity testing of these substances, and epidemiological study of exposed human populations, are needed for evaluating possible health hazards from hair dyeing.**

EXISTING methods for carcinogenicity testing of chemicals in animals are slow and expensive, and quite impracticable for monitoring the large numbers of new and existing environmental chemicals. There is consequently great current interest in various short-term assays that, though not capable of *proving* the carcinogenicity of a compound, may be valuable as screening tests.

Of the battery of tests considered necessary for adequate prescreening, at present the most useful appear to be those which detect chemically-induced reversion to prototropy in amino-acid-requiring mutants of *Salmonella typhimurium* and *Escherichia coli*. These test have been developed particularly by Ames and his colleagues, who have reported the results of testing some 300 carcinogenic and non-carcinogenic compounds (McCann *et al.*, 1975; McCann and Ames, 1976).

In June 1975, independent studies were reported by Ames, Kammen and Yamasaki and by Searle *et al.*, which showed that a range of hair-dye formulations, marketed in the U.S.A. and U.K. respectively, had considerable activity as mutagens in these bacterial tests. Various aromatic-amine constituents of the dyes were also found active. Shortly afterwards, MacPhee and Podger (1975) reported the mutagenicity of hair dyes sold in Australia. There has since been much discussion regarding the significance of these results, which brought to the fore questions concerning both the possible health hazards of exposure to these widely used dye chemicals, and the validity of the bacterial tests as indicators of carcinogenic activity.

The test carried out by Ames *et al.*, (1975) derived from an observation made during a students' practical class. Those reported by Searle *et al.*, (1976) were carried out as a result of animal tests of two proprietary hair dyes, to which our attention had been directed because of their use by a patient at the East Birmingham Hospital (Gyde, personal communication 1973).

The patient, a 52-year-old married woman, presented in 1972 with anaemia and neutropenia. Routine questioning elicited that she had frequently used two "semi-permanent" (non-oxidizing) hair dyes over several years. The anaemia responded to iron but, as expected, she remained neutropenic. Because of a suspicion that the hair dyes might have been a cause of her condition, she was advised to reduce her frequency of use, but apparently did not do so. In 1973 she developed acute myeloid leukaemia from which she died.

It was, of course, realized that the association of prolonged heavy hair-dye usage by this patient and her disease might be entirely fortuitous, but it was nevertheless thought desirable to carry out tests on the actual dyes used, for evidence of carcinogenicity in experimental animals. As these dyes are employed incorporated with detergent in a shampoo base, the only practicable means to test the complete preparations seemed to be by skin application to mice, and this was regarded as having at least some relevance to the conditions of human usage.

This communication reports the results of these experiments, during which there was a demonstration that the dyes were mutagenic in bacteria (Searle *et al.*, 1975). Preliminary accounts were presented to the British Association for Cancer Research (Searle, Harnden and Gyde, 1975) and elsewhere (Venitt and Searle, 1976; Searle, 1977).

### MATERIALS AND METHODS

*Hair dyes.*—These were Rimmel hair colourant shampoos of two shades, "Golden Silk" and "Really Brown" (referred to as GS and RB), purchased from a large chain store. The active ingredients were 2-nitro-p-phenylenediamine (2NPPD; Colour Index 76070) and 4-nitro-o-phenylenediamine (4NOPD; C.I. 76021) in GS, and C.I. Acid Black 107 (an azo-dye-metal complex) and 4-amino-2-nitrophenol (C.I. 76555) in RB.

GS and RB are "semi-permanent" dyes, which are used directly without addition of oxidant and are gradually removed from the hair during subsequent shampooings. They are dark viscous fluids, used in a similar manner to shampoos, but users are instructed to leave the second application on the hair for 10–15 min (GS) or 20 min (RB) for colouring to take place. The active constituents of GS and RB are believed to be present in many other proprietary products, the sole reason for testing these particular colourants being their use by the patient referred to above.

For application to mice, one volume was diluted with 4 parts of deionized water and 5 parts of acetone. Control mice received aqueous acetone (50% v/v).

It was realized that 4NOPD has found use as a reagent for $\alpha$-oxoacids and that some reaction of the diamines with acetone was possible. This was checked by thin-layer chromatography of the aqueous acetone dilutions of GS on Kieselgel containing fluorescer, using chloroform/ethanol (9/1, v/v) as solvent. 2NPPD and 4NOPD gave sharp spots of Rf 0·65 and 0·50 respectively. Non-UV-absorbing material, visualized by iodine vapour, remained at the origin, and was thought to be detergent. Heating the diluted solution at 60–65°C for 1 h produced a new faint brown spot at Rf 0·85, and a trace was also present after 3 weeks standing at room temperature. It is not, however, thought that any significant solvent interaction product was present in the solutions as applied to the mice. Nevertheless, after the first few weeks solutions were prepared shortly before use instead of each 1–2 weeks, and additional mice were added to the GS-treated groups.

*Animals.*—These were male and female mice of the albino A/Bcr and grey DBAf/Bcr strains, maintained in these laboratories by brother–sister mating for 23 and 13 years respectively. They were kept on sterilized sawdust in Makrolon boxes containing 5 animals and were fed modified rat/mouse breeding cube diet (Heygate, Ltd., Northampton) and tap water *ad libitum*.

*Treatment.*—Mice were first treated when 6–7 weeks old. Hair was removed from their backs with electric clippers before treatment and at intervals thereafter as required. The diluted dyes were applied to the clipped back skin using 0·5 ml glass pipettes. The volume applied was 0·4 ml per application, reduced to 0·2 ml at 24 weeks for all DBAf mice only, owing to toxic effects noted below in dye-treated animals. The applications, normally twice weekly, totalled 138 over the 80 weeks of the experiment. For human hair colouring, one 26 ml bottle is used on each occasion. Each application of GS or RB thus represented very approximately a 4-fold increase over the human application on a body-weight basis (2-fold when dose was reduced in DBAf mice).

Mice were killed for gross and histological examination if they showed evidence of tumours or became sick, or at 80 weeks from first treatment. Relatively few animals were lost to examination through unexpected death and autolysis, these being mostly GS-treated mice.

Tumours and other tissues for histological examination were fixed in formalin–acetic acid–methanol (1 : 1 : 8 by volume) and embedded in paraffin wax. Sections (5 $\mu$m) were stained with Harris' haematoxylin and eosin and with other stains when required.

*Statistical analysis.*—The statistical significances of the observed differences between control and treated groups were determined using the "logrank test" (Peto *et al.*, 1977). A survival time in weeks was determined for each mouse and in the context, of the analysis, an "event" was recorded only if death was accompanied by the diagnosis of a tumour. Deaths from other causes were, however, accounted for in the figure for "extent of exposure".

Taking each mouse-strain as a group, controls were compared with treated mice for all tumours and separately for lymphomas. Subsequently, comparisons in terms of strain, sex, and dye (8 tests in all) were made for "all tumours" alone, the numbers for lymphoma being too small to subdivide. To allow for the possible effect that sex might have on prognosis, the individual results for males and females were added to study the total effect.

### RESULTS

### *Toxicity*

The treatments were well tolerated by the A-strain mice and initially by the DBAf mice also. However, between 13 and 26 weeks of treatment some male DBAf mice became emaciated and were killed, and the volumes applied were therefore halved at 24 weeks in this strain only.

The toxic effects were centred on the urogenital tract, and may have been at least partly due to obstruction by crystals which were sometimes seen in the bladder and on the skin round the penis. The penile region was frequently distended and in 3 mice small squamous papillomas developed here. The bladder and seminal vesicles were sometimes very distended and, microscopically, dilation of renal tubules was seen. This evidence of toxicity was much less common in RB-treated mice, the times to 75% survival being 48 weeks (GS) and 64 weeks (RB). Of the DBAf controls, 78% survived to the end of the experiment at 80 weeks.

Many DBAf mice had noticeably distended stomachs at necropsy and histological examination showed chronic gastritis in 3 controls, 4 GS-treated and 9 RB-treated mice.

### *Tumour incidence by strain*

Sixty-four tumours were observed in the treated and control mice, and times at which they were found are summarized in Table I. Tumours of the lymphoid system accounted for half of those observed, and are listed separately. An initial analysis of the results showed a marked difference in the pattern of tumour development between strains. Each strain has therefore been considered separately.

When tumours at all sites were analysed for Strain A mice (Table II) the observed number of 13 tumours in controls was not significantly different from that of 29 tumours in the two treated groups ($x^2 = 0{\cdot}005$; d.f. $= 1$; $P > 0{\cdot}05$), nor was there a significant excess of lymphomas in the treated groups ($x^2 = 0{\cdot}005$; d.f. $= 1$; $P > 0{\cdot}05$). Further subdivision by sex and treatment group showed equally close correspondence between observed numbers and "extent of exposure".

For tumours at all sites in DBAf mice the difference between observed numbers for controls (3) and the treated group (19) was statistically significant ($x^2 = 6{\cdot}06$; d.f. $= 1$; $P < 0{\cdot}05$). Although in this strain only one lymphoma was observed in controls and 8 in the treated groups, the numbers were too small for numerical assessment.

### *Tumour incidence by treatment*

No statistically significant effect could be demonstrated in Strain A mice treated

TABLE I.—*Incidence of Tumours in A and DBAf Mice Treated by Repeated Applications of Two Semi-permanent Hair Dyes*

| | Mice treated | | | | Mice with tumours | | | |
|---|---|---|---|---|---|---|---|---|
| Strain | Treatment | Sex | No. | Examined at *post mortem* | Lymphomas (weeks) | Other tumours | Weeks | Mice tumour-free (weeks)* |
| A | Control | M | 16 | 16 | 77 80 | Hepatoma<br>Lung adenoma | 80<br>80 80 80 | 60 75 80 (8) |
| | Dye GS | F | 16 | 16 | 61 75 80 80 80 | Lung adenoma | 75 80 | 36 50 59 72 80 (5) |
| | | M | 26 | 25 | 57† 57 80 †80 | Hepatoma | 80† | 33 49 56 69 69 71<br>75 77 80 (9) |
| | Dye RB | F | 26 | 23 | 48 65 80 80 | Lung adenoma<br>Lung adenoma | 57† 79 80 80 80<br>51 78 80 80 | 46 55 66 72 78 80 (10) |
| | | M | 16 | 16 | 72 80 | Hepatoma | 80 | 41 44 71 75 75 79<br>80 (5) |
| | | F | 16 | 16 | 38 38 74 80 80 80 | Lung adenoma | 80 80 | 75 75 75 79 80 (6) |
| DBAf | Control | M | 16 | 15 | | Hepatoma | 80 | 48 65 70 71 80 (10) |
| | | F | 16 | 15 | 72 | Lung adenoma | 80 | 48 61 80 (11) |
| | Dye GS | M | 26 | 23 | 26 | Penile skin papilloma | 29 39 47 | 22 31 32 48 51 53<br>58 61 63 66 70 73<br>76 77 79 80 (3) |
| | | F | 22 | 18 | 37 41 73 80† | Ovarian cystadenoma<br>Uterine fibrosarcoma | 80† 80 80 80<br>66 69 | 59 73 78 79 80 (5) |
| | Dye RB | M | 17 | 16 | 47 | | | 13 19 50 53 64 66<br>67 69 70 80 (6) |
| | | F | 15 | 15 | 41 71 | Granulosa-cell tumour<br>Ovarian cystadenocarcinoma | 79<br>80 | 64 64 71 72 80 (7) |

* No. of tumour-free mice at 80 weeks in parentheses
† Mouse with additional primary tumour.

TABLE II.—*Tumour Incidence in A and DBAf Mice (Both hair dyes)*

| Strain | Site | | Group Control | Group Test | $x^2$ | *P* |
|---|---|---|---|---|---|---|
| A | All tumours | O | 13 | 29 | 0·005 | NS |
| | | E | 13·21 | 28·79 | | |
| | Lymphomas | O | 7 | 16 | 0·005 | NS |
| | | E | 7·16 | 15·84 | | |
| DBAf | All tumours | O | 3 | 19 | 6·06 | <0·05 |
| | | E | 8·64 | 13·36 | | |
| | Lymphomas | O | 1 | 8 | | |
| | | E | 3·24 | 5·76 | NC | — |

O = Observed number of tumours

E = Extent of exposure

*P* = Probability of the observed difference between control and test group being due to chance.

$$x^2 = \frac{(O_C - E_C)^2}{E_C} + \frac{(O_T - E_T)^2}{E_T}$$

NS = $P > 0{\cdot}05$

NC = $x^2$ not computed as E value too low.

TABLE III.—*Tumour Incidence in DBAf Mice Treated with Dye GS*

| Sex | | Group Control | Group Test | $x^2$ | *P* |
|---|---|---|---|---|---|
| Males | O | 1 | 5 | NC | — |
| | E | 3·32 | 2·86 | | |
| Females | O | 2 | 10 | 5·47 | < 0·05 |
| | E | 6·05 | 5·95 | | |
| Both | O | 3 | 15 | 9·03 | < 0·01 |
| | E | 9·37 | 8·63 | | |

with either GS or RB. In DBAf mice treated with RB the observed numbers were also very close to their expectations, but for those treated with GS the observed number was significantly different from that of controls ($x^2 = 9{\cdot}03$; d.f. $= 1$; $P < 0{\cdot}01$) (Table III). Although the number of tumours in males was too small for assessment, the direction of the difference was the same as in females, for which a significant effect was demonstrated ($x^2 = 5{\cdot}47$; d.f. $= 1$; $P < 0{\cdot}05$).

*Lymphoid tumours*

Although a significant excess of lymphoid tumours could not be demonstrated, they were first seen from 38 weeks in treated A mice and 26 weeks in DBAf. The earliest tumour in control mice was seen at week 61.

The lymphoid tumours consisted of small uniform basophilic cells, resembling the well-differentiated lymphocytic lymphoma of humans. However, these tumours appeared more malignant than the human equivalents. They were not confirmed to the lymph nodes, and extensive infiltration was seen in many organs, such as lung, liver, spleen and kidney, closely resembling the distribution of a leukaemic infiltrate (chronic lymphocytic leukaemia) in man. The renal infiltrate was often heavy and diffuse (Fig. 1) with extrarenal extension reminiscent of a leukaemic infiltrate. Lymphoma involving the spleen frequently caused loss of the normal splenic architecture (Fig. 2).

*Female reproductive-tract tumours*

These occurred in dye-treated DBAf mice only. Two mice in the GS group had uterine sarcomas. One, a pleomorphic fibrosarcoma in a mouse killed at 66 weeks, had become visible as a swelling in the genital region some 18 weeks earlier. The other, at 69 weeks, was a large, poorly differentiated fibrosarcoma which was widely infiltrating the uterus, with metastases in the liver and lungs. This tumour was composed of closely packed interwoven bundles of fusiform cells (Fig.3)

FIG. 1.—Female DBAf mouse, treated dye GS; 37 weeks. Heavy diffuse small-cell lymphomatous (leukaemia-like) infiltrate of kidney. Note extracapsular extension (right). H. & E. × 120.

FIG. 2.—Female A mouse, treated dye RB; 80 weeks. Splenic lymphoma showing replacement of normal architecture by sheets of small-cell lymphoma (lymphocytic lymphoma). Note multinucleated megakaryocyte (lower left). H. & E. × 300.

showing marked nuclear pleomorphism and frequent mitoses.

Six dye-treated DBAf mice developed ovarian tumours. At 80 weeks mucinous cystadenomas of the ovary were found in 4 GS-treated mice, in one case in addition to a lymphoma. These tumours were lined by complex infolded papillary epithelium composed of tall columnar cells with clear cytoplasm (Fig. 4). In the RB-treated mice an ovarian granulosa-cell tumour was found at 79 weeks and a cystadeno-

FIG. 3.—Female DBAf mouse, treated dye GS; 69 weeks. Uterine fibrosarcoma consisting of interlacing fascicles of fusiform cells infiltrating uterine muscle. H. &. E. × 120.

FIG. 4.—Female DBAf mouse, treated dye GS; 80 weeks. Mucinous cystadenoma of ovary showing typical tall columnar cells with clear cytoplasm. H. & E. × 120.

carcinoma at 80 weeks. This tumour had metastasized to the inguinal region and presented with a large mass consisting of well defined acinar structures set in a fibrous stroma (Fig. 5).

*Other tumours*

No more than a single hepatoma was found in any group. With one exception, all the lung tumours were in A mice. One in a GS-treated mouse was an adenocar-

FIG. 5.—Female DBAf mouse, treated dyeRB; 80 weeks. Metastatic ovarian cystadenocarcinoma in inguinal node. H. & E. × 120.

FIG. 6.—Male DBAf mouse, treated dye GS; 47 weeks. Squamous papilloma of penile skin. H. & E. × 300.

cinoma and one control lung tumour had metastasized, but the remainder were only small adenomas and there was no evident relationship to the treatments.

Three DBAf mice were observed with small squamous papillomas on and around the penis. These were all in the GS-treated group, and it seems more likely that these, and the associated toxic effects on the urogenital system, may have been caused by one or both of the nitrophenylenediamines or metabolites. The penile tumours were benign simple squamous papillomas consisting of hyperplastic basal cells and prickle cells (Fig. 6).

### DISCUSSION

These experiments were initiated because of a suspicion that frequent use of hair dyes might have been responsible for a case of acute myeloid leukaemia. It was thought that if either dye possessed reasonably strong carcinogenicity this might become evident, though it was realized that weak carcinogenicity would be difficult to demonstrate, particularly since any such action on organs other than the treated skin would depend on amounts of chemicals actually absorbed through the skin or ingested orally as a result of grooming.

As is seen from Table I, the main findings were tumours of the lymphoid system, which occurred in treated and control mice of both strains, and tumours of the reproductive tract, which were found in dye-treated DBAf females only.

In control A mice, the first lymphoid tumour was found at 61 weeks, while these occurred from 38 weeks in RB-treated animals and from 48 weeks with GS. The additional lymphoid tumours found on ending the experiment at 80 weeks, however, brought the final incidences to 25·0% and 21·9% in RB-treated and control animals respectively, and to 14·6% with GS treatment. The proportion of lymphoid tumours in controls was rather higher than expected from previous experience with this strain, for example in an experiment in which mice of 4 strains were treated neonatally with *N*-ethyl-*N*-nitrosourea (Searle and Jones, 1976) when only one lymphoid tumour occurred in 15 control A mice examined *post mortem*.

In the DBAf mice the experimental differences were more marked, with lymphoid tumours present in 5/31 (12·2%) of GS-treated mice and 3/31 (9·7% of RB-treated mice, compared with 1/30 (3·3%) in controls, despite longer survival in the latter group. Again, these tumours occurred considerably earlier with dye treatment, from 26 weeks with GS and 41 weeks with RB. Perhaps of more significance, however, were the tumours of the female reproductive tract which occurred towards the end of the experiment in dye-treated DBAf mice only. Two fibrosarcomas of the uterus, one with secondary deposits in the liver, were found at 66 and 69 weeks in GS-treated mice, and at 79–80 weeks 4 GS mice and 2 RB mice had ovarian tumours. Based on females examined *post mortem*, there were uterine or ovarian tumours in 6/18 (33·3%) GS mice and 2/15 (13·3%) RB mice. Ovarian tumours in the 4 GS-treated mice were cystadenomas. In the RB group a classical mucinous cystadenocarcinoma of the ovary had metastasized to an inguinal lymph node, the other ovarian tumour being a granulosa-cell tumour.

Also of interest in the DBAf strain were the toxic effects on the male urinary system, which in three instances were accompanied by small squamous papillomas of the skin near the penis. The observations were almost entirely confined to the GS group, and it seems likely that they depended on one or both of the nitrophenylenediamines or their metabolic products, even though the diluted solutions as applied contained the very low levels of approximately 0·06% (4NOPD) and 0·015% (2NPPD).

Summarizing these observations, it appears that the treatments of the Strain A mice resulted mainly in a small acceleration of the appearance of "spontaneous" lymphoid tumours. In DBAf mice, however, there was both an earlier appearance and an increased incidence of tumours. The excess was due mainly to uterine, ovarian and skin tumours which were not seen in the control group. Although no statistically significant excess of lymphomas could be demonstrated because of small numbers, the occurrence of one lymphoma in controls and 8 in the treated mice enhanced the total effect, indicating that the treatments appeared to have been carcinogenic for DBAf mice.

However, the applications were made using relatively complex mixtures and, with the possible exception of the skin papillomas, the tumours cannot with confidence be attributed to particular dye components of the colourant preparations. Unfortunately, true control solutions, containing detergent etc. but not the active ingredients, were not available. Though there was a specific reason for conducting the tests reported here, animal testing of whole proprietary colourants seems a generally unpromising approach because of the very large numbers of such products, their complex nature, and of severe limitations on the amounts which can be administered and on the practicable routes of administration. Firm evidence regarding the carcinogenicity of the various aromatic amino compounds used in hair colourants will have to come from separate tests of the individual chemicals.

The chief reason for suspecting that a number of hair dye constituents might be carcinogenic is their definite activity in a number of short-term screening tests, especially in the "Ames test" using bacterial mutants (Ames *et al.*, 1975; Searle *et al.*, 1975). Tests of some 300 carcinogens and non-carcinogens in the bacterial system showed a wide measure of qualitative agreement between mutagenic and carcinogenic activities (McCann *et al.*, 1975; McCann and Ames 1976) while in an important comparative study of 6 short-term tests for carcinogen detection (Purchase *et al.*, 1976) tests for bacterial mutagenicity and for cell transformation *in vitro* showed the most consistent correlations with the presence or absence of carcinogenic activity.

However, only in the case of 2,4-diaminotoluene (m-toluylenediamine) has reasonably firm evidence of carcinogenicity been reported to date from animal tests. This dye, now no longer used in hair dyes in the U.S.A. (Burnett *et al.*, 1975) was found to give rise to sarcomas on injection into rats (Umeda, 1955) and to liver carcinomas on feeding to rats (Ito *et al.*, 1969). It was recently claimed to be non-carcinogenic when applied to mouse skin (Giles, Chung and Kommineni, 1976) but this conclusion has been sharply criticized by Bridges and Green (1976) because of the small numbers of animals in the individual groups and the high incidence of tumours in control animals. They noted that overall there was a greater incidence of tumours in treated animals than in controls, and pointed out that failure to demonstrate a statistically significant increase in tumour yield does not necessarily justify a claim of non-carcinogenicity.

A very important factor in carcinogenicity testing of environmental materials is that of the dose level to be administered. Widely divergent views have been expressed on this. Reporting on the effects of applying mixtures of hair-dye components to mouse skin, Burnett *et al.* (1975) list reasons why they consider product-evaluation studies must involve conditions of use to obtain meaningful results. An advisory committee of the U.S. Food and Drugs Administration reporting on carcinogenicity testing of food additives and pesticides (1971), however, considered that tests should be carried out using doses and conditions likely to yield maximum tumour incidence. This will often mean the use of dosages several orders of magnitude above the levels encountered by man. Weisburger (1976) has stressed the importance of establishing maximum tolerated doses at an early stage in carcinogenicity testing, and pointed out that even with the firmly established human carcinogen 2-naphthylamine high doses were necessary to reproduce the human condition in animals.

The view expressed by Burnett *et al.* (1975) appears at first sight quite reasonable. However, having regard to factors such as the need to recognize weak as well as strong carcinogens and the common occurrence of spontaneous tumours in control animals, it seems clear that carcinogenicity tests which are limited to environmental levels are, on any practicable scale, incapable of giving assurances of safety of products used by millions of people. Moreover, when complex formulations are employed, as in the experiments of Burnett *et al.* (1975) and Kinkel and Holzmann (1973), as well as in the experiments reported here, it may be impossible to decide which substance is responsible for any effect that is detected without further prolonged experimentation. It is, of course, because of the many difficulties involved in testing large numbers of environmental materials that there is such great current interest in the various short-term screening tests (Montesano, Bartsch and Tomatis, 1976; Purchase *et al.*, 1976).

A further and more direct approach to obtaining information on possible adverse effects of environmental materials becomes practicable when, as in the case of hair dyes, they have had widespread use for one or more decades. This is, of course, to conduct epidemiological studies directly on the exposed human populations. As persons engaged in dyeing in the hair-dressing trade are exposed to considerably greater levels than are individual home dyers, these represent the population which it is probably most useful to study, and this in now being done in this country. National mortality trends for some major cancers are sometimes quoted (*e.g.* Corbett, 1976) as failing to provide evidence for a carcinogenic effect of hair dyes. Experience with important occupational carcinogens such as 2-naphthylamine suggests, however, that such data could only reasonably be expected to reveal a massive increase in a common cancer or the induction of a normally very rare one, and that valid epidemiological evidence is only likely to come from properly planned and conducted comparisons of exposed and non-exposed populations.

A number of aromatic amines used in hair dyes are well-known sensitizing agents (Hunter, 1975) and oxidizing (permanent) hair dyes are marketed with warnings that patch tests for sensitivity should be carried out before use. It has recently been suggested that some cases of aplastic anaemia may have resulted from hair-dye usage (Hamilton and Sheridan, 1976; Hans, 1976) though this view was contested by Jouhar (1976). This is a further area in which proper epidemiological study is necessary before we can expect to have a comprehensive picture of the health aspects of the present very large-scale usage of hair dyes.

We are very grateful to Mrs M. P. Prior for carrying out the statistical analysis of our results. We thank Miss V. Nash and Mr D. Sammons for their efficient technical assistance, Professor D. G. Harnden, Dr O. H. B. Gyde and Dr. S. Venitt for valuable discussions, and the Cancer Research Campaign for financial support of C.E.S.

### REFERENCES

AMES, B. N., KAMMEN, H. O. & YAMASAKI, E. (1975) Hair dyes are Mutagenic: Identification of a Variety of Mutagenic Ingredients. *Proc. natn. Acad. Sci. USA*, **72**, 2423.

BRIDGES, B. A. & GREEN, M. H. L. (1976) Carcinogenicity of Hair Dyes by Skin Painting in Mice. *J. Toxicol. environ. Hlth.*, **2**, 251.

BURNETT, C., LANMAN, B., GIOVACCHINI, R., WOLCOTT, G., SCALA, R. & KEPLINGER, M. (1975) Chronic Toxicity Studies on Oxidation Hair Dyes. *Fd. Cosmet. Toxicol.*, **13**, 353.

CORBETT, J. F. (1976) Hair dyes—their Chemistry and Toxicology. *Cosmet. Toiletries*, **91**, 21.

FOOD AND DRUGS ADMINISTRATION Advisory Committee on Protocols for Safety Evaluation. (1971).Panel on Carcinogenesis Report on Cancer Testing in the Safety Evaluation of Food Additives and Pesticides. *Toxicol. appl. Pharmacol.*, **20**, 419.

GILES, A. L., JR, CHUNG, C. W. & KOMMINENI, C. (1976) Dermal Carcinogenicity Studies by Mouse-skin Painting with 2,4-toluenediamine alone or in Representative Hair Dye Formulations. *J. Toxicol. environ. Hlth.*, **1**, 433.

HAMILTON, S. & SHERIDAN, J. (1976) Aplastic Anaemia and Hair Dye. *Br. med. J.*, **i**, 834.

HANS, R. J. (1976) Aplastic Anaemia and Hair Dye. *Br. med. J.*, **ii**, 422.

HUNTER, D. (1975) *The Diseases of Occupations.* 5th Edn. London: English Universities Press, p. 533.

ITO, N., HIASA, Y., KONISHI, Y. & MARUGAMI, M. (1969) The Development of Carcinoma in Liver of Rats treated with m-Toluylenediamine and Synergistic and Antagonistic Effects of other Chemicals. *Cancer Res.*, **29**, 1137.

JOUHAR, A. J. (1976) Aplastic Anaemia and Hair Dye. *Br. med. J.*, **i**, 1074.

KINKEL, H. J. & HOLZMANN, S. (1973) Study of Long-term Percutaneous Toxicity and Carcinogenicity of Hair Dyes (Oxidising Dyes) in Rats. *Fd. Cosmet. Toxicol.*, **11**, 641.

MCCANN, J. & AMES, B. N. (1976) Detection of Carcinogens as Mutagens in the *Salmonella*/ microsome Test: Assay of 300 Chemicals: Discussion. *Proc. natn. Acad. Sci. USA*, **73**, 950.

MCCANN, J., CHOI, E., YAMASAKI, E. & AMES, B. N. (1975) Detection of Carcinogens in the *Salmonella*/

microsome Test: Assay of 300 chemicals. *Proc. natn. Acad. Sci. USA*, **72**, 5135.

MacPhee, D. G. & Podger, D. M. (1975) Hair Dyes, *Med. J. Aust.*, **2**, 32.

Montesano, R., Bartsch, H. & Tomatis, L., (Eds.) (1976) *Screening Tests in Chemical Carcinogenesis.* Lyon: I.A.R.C. Sci. Publ. **12.**

Peto, R., Pike, M. C., Armitage, P., Breslow, N. E., Cox, D. R., Howard, S. V., Mantel, N., McPherson, K., Peto, J. & Smith, P. G. (1977) Design and Analysis of Randomised Clinical Trials Requiring Prolonged Observation of Each Patient. *Br. J. Cancer*, **35**, 1.

Purchase, I. F. H., Longstaff, E., Ashby, J., Styles, J. A., Anderson, D., Lefevre, P. A. & Westwood, F. R. (1976) Evaluation of Six Short Term Tests for Detecting Organic Chemical Carcinogens and Recommendations for their Use. *Nature, Lond.*, **264**, 624.

Searle, C. E. (1977) Evidence regarding the Possible Carcinogenicity of Mutagenic Hair Dyes and Constituents. *Colloques internat. CNRS*, **256,** 407.

Searle, C. E., Harnden, D. G. & Gyde, O. H. B. (1975) Tests of Two Hair Colourants for Carcinogenicity by Repeated Application to Mouse Skin (Abstract). *Br. J. Cancer*, **32**, 251.

Searle, C. E., Harnden, D. G., Venitt, S. & Gyde, O. H. B. (1975) Carcinogenicity and Mutagenicity Tests of Some Hair Colourants and Constituents. *Nature, Lond.*, **255**, 506.

Searle, C. E. & Jones, E. L. (1976) The Multipotential Carcinogenic Action of *N*-ethyl-*N*-nitrosourea Administered Neonatally to Mice. *Br. J. Cancer*, **33**, 612.

Umeda, M. (1955) Production of Rat Sarcoma by Injections of Propylene Glycol Solution of m-toluylenediamine. *Gann*, **46**, 597.

Venitt, S. & Searle, C. E. (1976) Mutagenicity and Possible Carcinogenicity of Hair Colourants and Constituents. *INSERM Symposia Ser.* **52**/*IARC Sci. Publ.*, **13**, 263.

Weisburger, J. H. (1976) In *Chemical Carcinogens*, Ed. C. E. Searle, Washington: Am. Chem. Soc. *Monogr. Ser.*, **173**, 13.